# Daily cigarette smoking among inpatients for substance use disorders in France, 2010–2020: Commonalities and specificities across substances

**DOI:** 10.18332/tid/194097

**Published:** 2024-11-05

**Authors:** Eric Janssen, Mike Vuolo, Guillaume Airagnes

**Affiliations:** 1 The French Monitoring Centre for Drugs and Drug Addiction, Paris, France; 2 Department of Sociology, The Ohio State University, Columbus, United States; 3 Unité de Formation et de Recherche de Médecine, Faculté de Santé, l’Assistance Publique – Hôpitaux de Paris, Centre - Université Paris Cité, Paris, France; 4 Population-based Cohorts Unit, The French National Institute of Health and Medical Research, Paris, France

**Keywords:** cigarette smoking, patients, substance use disorders, France

## Abstract

**INTRODUCTION:**

This study aims to estimate the prevalence and factors associated with cigarette smoking among patients in treatment for substance use disorders (SUD) in France.

**METHODS:**

We analyze a nation-wide dataset retrieving information on patients entering treatment for alcohol, opioid and stimulant use disorders between 2010 and 2020. We conduct multilevel Poisson regressions to determine the main factors associated with daily cigarette smoking among all patients who entered treatment for alcohol (n=607122), opioid (n=283381) or stimulant (n=57189) use disorders, and zero-truncated negative-binomial regressions to predict the average number of cigarettes per day.

**RESULTS:**

Daily cigarette smoking remains a widespread behavior among patients with SUD (overall prevalence: 72.2%, 95% CI: 72.1–72.3), with lower prevalence of daily cigarette smoking among patients treated for alcohol use disorders (69.9%, 95% CI: 69.8–70.0), and higher for patients treated for opioid (78.8%, 95% CI: 78.6–79.0) or stimulant use disorders (75.8%, 95% CI: 75.4–76.2). There was an overall increase in daily cigarette smoking over time (69.9%, 95% CI: 69.8–70.0 in 2010 vs 76.8%, 95% CI: 76.5–76.9 in 2020); however, the average number of cigarettes per day decreased (17.8 per day, 95% CI: 17.7–17.9 in 2010 vs 16.3 per day, 95% CI: 16.2–16.4 in 2020). The higher the education level, the fewer number of cigarettes per day; conversely, the higher the occupational status, the higher the number of cigarettes.

**CONCLUSIONS:**

The high prevalence of smoking among patients treated for SUD in France departs from the decreasing trend observed in the general population and remains a source of concern. It is necessary to implement tailored prevention strategies that target specific patient subgroups and increase staff awareness.

## INTRODUCTION

Tobacco use is the leading cause of avoidable deaths worldwide^1^, and is a major risk factor for cardiovascular and respiratory diseases, strokes and over 20 different types of cancer. In France, each year more than 75000 deaths are estimated as attributable to tobacco, mostly due to cigarette smoking^2^. Similar to other Western countries, prevention efforts over the last few decades have focused on the general population, with somewhat encouraging results: tobacco experimentation has receded steadily since 2000, among both adults^3^ and adolescents^4^. However, daily tobacco use remains relatively high, affecting more than three in ten adults aged 18–75 years^3^, and one in six participants aged 17 years^4^.

To date, the well documented decrease in tobacco use and cigarette smoking in the general population is not reflected in specific populations, such as patients under treatment for alcohol or substance use disorders (AUD/SUD). Compared with other adults, people with AUD and SUD consistently report higher levels of cigarette smoking^5^. There is additional evidence that patients continue to smoke during treatment, even when abstaining from substances^6^, raising specific concern of risks for somatic health problems that increase with age, including preventable diseases associated with tobacco smoking. Recent studies suggest that smoking cigarettes is common among patients in treatment, with prevalence ranging from 60% to 90%^7,8^. A metanalysis on cigarette smoking in addiction treatment centers across 20 countries suggested an overall prevalence of 84%^9^, with significant differences in smoking rates by substance-specific treatment: patients treated for opioid use disorders were more prone to be currents smokers than those for alcohol disorders. High levels of smoking were detected among a sample of patients entering treatment for substance use disorders in Norway (>93%) and the majority were still smoking one year after (69% of inpatients and 87% of patients on opioid maintenance treatment)^10^. By contrast, in the US, a recent study among adults with SUD receiving treatment, suggested a decreasing prevalence between 2006 (46.5%) and 2019 (35.8%)^11^, somewhat lower than prevalence measured in a prior study: in 2014, current smoking prevalence was 64.3% among patients with AUD and 55.0% among SUD^12^. A longitudinal study conducted in the UK measured baseline prevalence at 48.7%, and up to 61.0% in the case of patients treated for opioid disorders^13^.

Interestingly, few studies provide insights on cigarette use according to patients’ sociodemographic characteristics^5^. In general, male patients are more likely to be current and heavy smokers than female, as are younger patients. Cigarette smoking is more common among patients with psychiatric dsorders^14^. Similar to adults in the general population, outpatients are sensitive to a wide range of external influence, with significant impact of marketing and advertising^15^. A recent study suggests that patients are sensitive to exposure to staff smoking^8^, an overlooked issue given that prevalence of smoking among physicians is high, around 21%^16^. An increased likelihood of smoking has also been found among people who use drugs involved in the criminal justice system or with criminal experience^17^.

Despite converging evidence of the related negative health outcomes of concurrent smoking during treatment, data on that particular matter in France are scant. In a study of patients with alcohol disorders, the prevalence of current smokers was estimated to be 82%^18^, a rate somewhat below those in prior studies conducted in the 1990s^19^. To our knowledge, no study has provided further information since, and none has provided figures on cigarette use among patients treated for other substance disorders. The absence of new data is more surprising in the context of significant reduction in cigarette smoking in the general population during the past decade^20^. In the absence of sufficient evidence, this study has two main objectives: 1) to provide updated estimates of smoking prevalence among patients treated for substance use disorders and assess and substance-specific prevalence among patients in France; and 2) to assess factors related to cigarette smoking.

## METHODS

### Data

The data come from a yearly updated compendium on addictions and treatments (*Recueil commun sur les addictions et les prises en charge* - RECAP), carried out at the national level in France^21^. Treatment centers in France are State-funded, medically-driven entities aiming at total cessation. Treatment centers provide free service regardless of age, gender, income or race. Typically, treatment centers also include psychological and social support. Note that until 2010, alcohol disorders and illicit substance misuse were treated separately, each in devoted premises. However, in 2011, the Social Security released a unified protocol stipulating that all treatment centers were to provide services to people who use substances, regardless of the nature of the substance involved.

In their annual activity reports, all treatment centers are requested to provide data on all patients welcomed into their premises during a full calendar year, following the European protocol for registering treatment demands, one of the European Monitoring Centre for Drugs and Drug Addiction’s (EMCDDA) key indicators. Each year, 80% of treatment centers on average provide data; among those providing data, 100% of patients are included. The face-to-face, standardized questionnaire includes information on individual substance use (frequency of use, route of administration, age at onset and an assessment of the severity of use), health and sociodemographic characteristics^21^. The information is updated each time a patient consults with a staff member. The survey was approved by an internal steering committee which acts as the equivalent of an Institutional Review Board, and by the National Data Protection Authority (CNIL).

### Participants

In the present study, we focus on families of substances, namely patients who entered treatment for alcohol use disorders (AUD, n=607122), opioid use disorders (OUD: heroin, fentanyl and others, n=283381), or stimulant use disorders (StUD: powder cocaine, crack cocaine, ecstasy/MDMA, (meth)amphetamines, synthetic cathinones, others, n=57189) (see Supplementary file Table 1 for details on the samples). Following the EMCDDA’s recommendations^22^, patients aged 15–64 years were included.

### Smoking status

We are interested in two dependent variables. The first dependent variable measures whether a patient smoked cigarettes during the past 30 days (binary variable). The second variable takes the following form: ‘If you smoked during the past 30 days, on average how many cigarettes a day did you smoke?’, with potential responses ranging from 0 up to 99 cigarettes (continuous variable).

### Covariates

Covariables include year of survey (from 2010 to 2020, with 2010 as reference), gender (male as reference) and age (15–24, 25–34, and 35–64 years as reference). We retain two variables to approximate sociodemographic status: education level (incomplete secondary education as reference; high school diploma; some college or college completed) and the socioeconomic status assessed by mean of the occupation, following the official typology of the National Institute of Statistics and Economic Studies^23^. The 9-item response was recoded in a simplified 3-category variable: low position (inactive, pensioner) as reference; intermediate position (farmer, blue collars, clerks); and high position (executive, managers, liberal professions).

Other substance-related questions include the age of initiation of both tobacco and the substance leading to treatment (aged <15 years; and ≥16 years as reference), an assessment of the severity of the substance disorder (high severity as reference; low or mild severity), psychiatric disorder as assessed by the medical and psychological staff [none as reference; depression or anxiety syndrome; other syndrome (psychotic or delusional syndrome, personality disorders, behavioral syndrome, eating disorders)], and the duration elapsed since beginning of treatment (1–5 months as reference; 6–11 months; and ≥12 months)^24^.

### Statistical analysis

Descriptive statistics include prevalences and their associated percentile bootstrapped 95% confidence limits (100 replications) and Cochrane-Armitage test for time trends (in sensitivity analysis, the Mann-Kendall test was used and led to similar results). Given the hierarchical structure of the data, in which patients are nested in treatment centers (i.e. clustering effect adding an additional layer of dependence among observations), we first conducted multilevel, random intercept modified Poisson regressions with corrected standard errors^25^ to identify factors associated with cigarette smoking. In order to numerically assess the clustering effect, we calculated the intraclass correlation coefficient (ICC) and the median rate ratio (MRR), an extension of the median odds ratio (MOR) for count variables that translates the higher-level variance into the incidence ratio scale. Stated more practically, the MMR shows the extent to which the individual-level probability varies across clusters. For a clearer interpretation, we show predicted probabilities for these three measures, obtained via the margins command in Stata^®^ 17.1 software^21^.

Next, we analyzed factors associated with the number of cigarettes smoked daily. Given that in our particular case, occasional cigarette smoking was not considered, e.g. all cigarette smokers in our study are daily smokers, we used a zero-truncated negative binomial regression, accounting for extra variance in the response variable. Note that an ancillary analysis considered alternative modeling (multilevel multinomial logistic regressions studying: non-smokers; moderate smokers, 1–9 cigarettes per day; and intensive smokers, ≥10 cigarettes per day), showing an extremely marginal effect of the second (treatment) level. Thus, we refer to single level zero-truncated models with cluster-controlled standard errors. Parameters with two-tailed p<0.05 were considered statistically significant.

## RESULTS

The prevalence and mean number of cigarettes per day are shown in Table 1. Patients with opioid disorders show the highest cigarette smoking prevalence, followed by those treated for stimulants, and alcohol disorders (e.g. 86.8%, 95% CI: 86.7–86.9; 80.5%, 95% CI: 80.4–80.6; and 73.2%, 95% CI: 73.1–73.3, in 2020, respectively). Conversely, patients with AUD have a higher number of cigarettes, compared with OUD and StUD patients (e.g. mean of 16.6 cigarettes per day, 95% CI: 16.5–16.7; 15.8, 95% CI: 15.7–15.9; and 15.1, 95% CI: 15.0–15.2 in 2020, respectively). In all three cases, the data reveal a mixed process: the increase of past month cigarette smoking over time (5.1% for AUD, 23.6% for OUD, 16.5% for StUD) has been accompanied with an overall 10% decrease in the average number of cigarettes smoked daily.

**Table 1:** Smoking prevalence among patients treated for SUD and average number of cigarettes per day, 2010–2020

*Year*	*Alcohol (N=607122)*				*Opioids (N=283381)*				*Stimulants (N=57189)*			
	*%*	*95% CI*	*Mean ^a^*	*95% CI*	*%*	*95% CI*	*Mean ^a^*	*95% CI*	*%*	*95% CI*	*Mean ^a^*	*95% CI*
2010	69.6	68.8–70.4	18.4	18.3–18.6	70.2	69.6–70.7	17.6	17.5–17.7	69.1	67.6–70.6	16.9	16.6–17.2
2011	65.7	65.1–66.2	18.6	18.5–18.7	71.8	71.3–72.3	17.6	17.4–17.7	69.0	67.5–70.5	16.8	16.5–17.1
2012	64.7	64.2–65.2	18.3	18.2–18.4	72.3	71.8–72.8	17.1	16.9–17.2	64.9	63.3–66.4	16.4	16.1–16.7
2013	67.3	66.9–67.6	18.3	18.2–18.3	73.6	73.1–74.1	17.0	16.9–17.1	68.7	67.3–70.0	16.8	16.5–17.0
2014	65.4	65.0–65.8	17.9	17.8–18.0	74.5	74.0–75.1	16.8	16.7–16.9	70.5	69.0–71.9	16.2	15.9–16.4
2015	64.9	64.6–65.3	17.8	17.8–17.9	76.4	75.9–76.9	16.7	16.6–16.8	67.9	66.6–69.3	16.0	15.7–16.2
2016	69.4	69.0–69.7	17.5	17.4–17.6	82.2	81.7–82.7	16.5	16.4–16.6	76.9	75.7–78.1	15.8	15.6–16.0
2017	74.0	73.6–74.3	17.2	17.1–17.2	88.0	87.6–88.3	16.3	16.2–16.4	82.3	81.3–83.2	15.7	15.5–15.9
2018	73.6	73.3–73.9	17.1	17.0–17.2	87.9	87.5–88.3	16.2	16.1–16.3	83.3	82.5–84.2	15.5	15.3–15.7
2019	73.0	72.7–73.4	16.9	16.8–16.9	86.8	86.3–87.2	16.1	16.0–16.2	80.9	80.0–81.7	15.4	15.2–15.5
2020	73.2	72.9–73.5	16.6	16.5–16.7	86.8	86.4–87.2	15.8	15.7–15.9	80.5	79.6–81.4	15.1	15.0–15.3
%Δ_2010-20_	5.1 ***		-10.0		23.6 ***		-10.2		16.5 ***		-10.3	

a Mean number of cigarettes per day. Cochrane-Armitage test for trends,

*** p<0.001. Source: RECAP survey.

The results of the multivariable analysis on cigarette smoking are shown in Table 2. The increasing trend over time is confirmed in all three cases: compared to 2010, AUD, OUD, and StUD patients are 20%, 32% and 29% more likely to smoke in 2020, respectively. Females treated for AUD are less likely to smoke (incidence rate ratio, IRR=0.95; 95% CI: 0.94–0.96), as are those treated for OUD (IRR=0.99; 95% CI: 0.95–1.00), but more prone when treated for StUD (IRR=1.02; 95% CI=1.00–1.03). Smoking is more common among older patients in all three cases. In the case of patients treated for AUD, relative to those aged 15–24 years, the associated risks are 8% higher for those aged 25–34 years and 4% higher for those aged 35–64 years. A similar tendency, albeit less pronounced, occurs in the case of patients treated for OUD (2% and 3% higher, respectively) and StUD (3% and 2% higher, respectively). Smoking is also related to education level, with patients with a college degree having consistently lower risks compared to those with incomplete secondary education (-4%, -3%, -4% for AUD, OUD, and StUD, respectively). Occupational status does not appear to be associated with smoking, except in the instance of higher versus lower status for AUD (-3%). Early age of initiation of the substance triggering treatment is associated with smoking in the case of AUD (IRR=1.06, 95% CI: 1.04–1.07) and OUD (IRR=1.01, 95% CI: 1.00–1.03). The lower is the severity of AUD and OUD, the higher is the likelihood to smoke (3% and 2%), whereas it is lower for patients treated for StUD (-2%). Smoking is more common for patients on treatment for a year or more (11%, 6% and 5% for AUD, OUD, and StUD, respectively), and for those with depression or anxiety disorders (11% for AUD, 9% for OUD and StUD), and even higher in case of other psychiatric disorders (15% in all three cases). The MRR is 1.27 (95% CI: 1.22–1.31), 1.43 (95% CI: 1.33–1.52) and 1.37 (95% CI: 1.30–1.44) for AUD, OUD, and StUD, respectively, with 95% confidence limits that exclude the value 1, denoting significant between-cluster variance. The significant between-cluster heterogeneity suggests that patients’ smoking is center-dependent.

**Table 2:** Multilevel modified Poisson regression of risk factors for cigarette smoking among patients treated for SUD, 2010–2020

*Variables*	*Categories*	*Alcohol*		*Opioids*		*Stimulants*	
		*IRR*	*95% CI ^a^*	*IRR*	*95% CI ^a^*	*IRR*	*95% CI ^a^*
**Year of survey**	2011	0.99	0.93–1.06	1.02	0.99–1.04	1.00	0.97–1.04
	2012	1.01	0.95–1.07	1.02	0.99–1.06	0.94*	0.90–0.99
	2013	1.04	0.97–1.11	1.04	0.99–1.09	0.99	0.94–1.04
	2014	1.08*	1.00–1.15	1.07*	1.00–1.13	1.04	0.97–1.12
	2015	1.04	0.97–1.11	1.09**	1.03–1.15	1.00	0.93–1.08
	2016	1.14***	1.06–1.23	1.23***	1.14–1.33	1.21***	1.10–1.33
	2017	1.23***	1.14–1.32	1.35***	1.25–1.45	1.32***	1.20–1.45
	2018	1.23***	1.15–1.32	1.35***	1.24–1.45	1.35***	1.21–1.50
	2019	1.21***	1.12–1.31	1.33***	1.23–1.44	1.31***	1.17–1.46
	2020	1.20***	1.12–1.29	1.32***	1.22–1.42	1.29***	1.16–1.44
**Gender**	Female	0.95***	0.94–0.96	0.99*	0.98–1.00	1.02*	1.00–1.03
**Age** (years)	25–34	1.08***	1.06–1.09	1.02*	1.00–1.04	1.03*	1.00–1.05
	35–64	1.04***	1.02–1.06	1.03*	1.00–1.05	1.02*	1.00–1.05
**Education level**	High school diploma	0.96***	0.95–0.97	0.99	0.98–1.00	1.00	0.99–1.01
	Some college	0.96***	0.95–0.97	0.97***	0.96–0.99	0.96***	0.94–0.98
**Occupational category**	Intermediate	1.00	0.99–1.01	1.00	0.99–1.01	1.00	0.98–1.01
	Higher	0.97***	0.96–0.98	0.99	0.97–1.01	0.98	0.96–1.01
**Age initiation of SUD**	Before the age of 15 years	1.06***	1.04–1.07	1.01*	1.00–1.03	1.02	0.99–1.04
**SUD severity assessment**	No dependence	1.03***	1.01–1.04	1.02***	1.01–1.03	0.98*	0.97–1.00
**Length of treatment**	6–11 months	1.01**	1.00–1.02	1.01	1.00–1.02	1.00	0.99–1.01
	>11 months	1.11***	1.09–1.13	1.06***	1.04–1.08	1.05***	1.04–1.07
**Psychiatric disorders**	Anxiety or depression	1.11***	1.09–1.13	1.09***	1.07–1.12	1.09***	1.08–1.11
	Other disorder	1.15***	1.12–1.17	1.15***	1.12–1.18	1.15***	1.12–1.19
	Treatment center variance	1.07***	1.04–1.09	1.15***	1.09–1.22	1.12***	1.08–1.16
	Observations	606904		283337		57155	
	ICC	0.06		0.14		0.11	
	MRR	1.27	1.22–1.31	1.43	1.33–1.52	1.37	1.30–1.44

IRR: incidence rate ratio; represents the increased rate ratio per 1 unit increase in the scale. ICC: intraclass correlation coefficient. MRR: median rate ratio.

*** p<0.001.

** p<0.01.

* p<0.05.

a Robust 95% CI. Source: RECAP survey.

**Figure 1:**
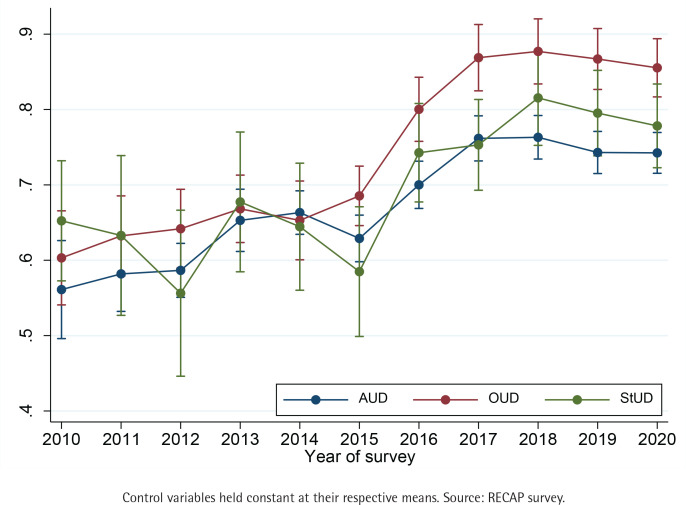
Probabilities (with 95% CI) of daily smoking for patients treated for substance use disorders, 2010–2020

For a clearer depiction of the prior results, we calculated the probabilities of daily smoking for each year, with other variables held constant at their respective means (Figure 1). We note that the prior results hold, with a pronounced effect in the increasing trend until 2017. In all cases afterwards, the probabilities of daily smoking have remained stable, albeit at high level (p>0.7 of AUD and p>0.8 for OUD and StUD in 2020).

The results of the multivariable analysis on the number of cigarettes per day are shown in Table 3. The decreasing trend across time observed in the descriptive results is confirmed: in 2020, patients treated for AUD smoked on average 11% fewer cigarettes than in 2010, 12% fewer for OUD patients, and 10% fewer for patients treated for StUD. In all three cases, females smoke fewer cigarettes (-2% for AUD and StUD, -3% for OUD), whereas older patients smoke more. This result is mirrored by the positive effect of early initiation to tobacco: among patients treated for AUD, tobacco initiation before the age of 15 years is associated with an 8% increase in the average number of cigarettes smoked. A similar trend is observed, to a less extent, with early initiation to the substance inducing treatment (2%, 2% and 4% for AUD, OUD, and StUD, respectively). Interestingly, both education level and occupational category are here strongly associated with the average number of cigarettes in all cases, although in opposite manner: the higher the education level, the fewer the number of cigarettes per day, whereas higher occupational category yields a significantly increase. The effect of length of treatment is unclear: intermediate duration (6–11 months) tends to reduce the average of cigarettes per day among patients with AUD and StUD (-1% and -3%), as opposed to longer duration, suggesting a 1% increase in the case of AUD and OUD. Finally, psychiatric disorders are strongly related to the number of daily cigarettes, with a 3–5% increase regardless of the type of the diagnosed comorbidity.

**Table 3:** Zero-truncated negative binomial regression of risk factors for the number of cigarettes smoked per day during the past 30 days among patients treated for SUD, 2010–2020

*Variables*	*Categories*	*Alcohol*		*Opioids*		*Stimulants*	
		*IRR*	*95% CI ^a^*	*IRR*	*95% CI ^a^*	*IRR*	*95% CI ^a^*
**Year of survey**	2011	1.01	0.99–1.02	0.99	0.98–1.00	0.99	0.96–1.02
	2012	0.98*	0.96–1.00	0.96***	0.95–0.98	0.96*	0.93–1.00
	2013	0.96***	0.94–0.98	0.94***	0.92–0.96	0.96	0.92–1.01
	2014	0.96***	0.94–0.98	0.94***	0.93–0.96	0.95*	0.91–1.00
	2015	0.95***	0.93–0.97	0.93***	0.92–0.95	0.94**	0.90–0.98
	2016	0.94***	0.92–0.96	0.93***	0.91–0.94	0.94**	0.91–0.98
	2017	0.92***	0.90–0.94	0.91***	0.90–0.92	0.93***	0.90–0.97
	2018	0.91***	0.89–0.93	0.90***	0.89–0.92	0.92***	0.89–0.96
	2019	0.90***	0.89–0.92	0.90***	0.88–0.91	0.92***	0.88–0.95
	2020	0.89***	0.87–0.91	0.88***	0.86–0.89	0.90***	0.87–0.94
**Gender**	Female	0.98***	0.97–0.99	0.97***	0.96–0.98	0.98*	0.96–1.00
**Age** (years)	25–34	1.12***	1.11–1.14	1.09***	1.08–1.11	1.09***	1.07–1.11
	35–64	1.25***	1.23–1.26	1.16***	1.14–1.18	1.13***	1.11–1.15
**Education level**	High school diploma	0.98***	0.98–0.99	0.97***	0.96–0.98	0.96***	0.95–0.97
	Some college	0.96***	0.95–0.97	0.96***	0.94–0.97	0.92***	0.89–0.94
**Occupational category**	Intermediate	1.03***	1.02–1.03	1.00	0.99–1.01	1.03***	1.01–1.04
	High	1.07***	1.06–1.08	1.03***	1.02–1.05	1.08***	1.05–1.10
**Age initiation of cigarettes**	Before the age of 15 years	1.08***	1.07–1.09	1.04***	1.03–1.06	1.05***	1.03–1.08
**Age initiation of SUD**	Before the age of 15 years	1.02***	1.01–1.02	1.02**	1.01–1.04	1.04**	1.02–1.07
**SUD severity assessment**	No dependence	0.97***	0.96–0.97	1.00	0.99–1.01	0.98**	0.97–1.00
**Length of treatment**	6–11 months	0.99***	0.98–0.99	0.99	0.98–1.00	0.97***	0.96–0.98
	>11 months	1.01***	1.01–1.02	1.01*	1.00–1.02	1.00	0.98–1.01
**Psychiatric disorders**	Anxiety or depression	1.05***	1.04–1.06	1.03***	1.02–1.05	1.05***	1.03–1.07
	Other disorder	1.03***	1.03–1.04	1.03***	1.02–1.04	1.05***	1.03–1.07
	Alpha^b^	0.12***	0.11–0.13	0.12***	0.12–0.13	0.12***	0.11–0.14
	Observations	422533		223190		43344	

a Robust 95% CI.

b Alpha: a measure and test of overdispersion (the variance is greater than the mean, supporting the use of a zero-truncated negative binomial over a Poisson regression).

*** p<0.001,

** p<0.01,

* p<0.05, Source: RECAP survey.

## DISCUSSION

Taking advantage of a nationwide, standardized survey, this study provided updated prevalence of cigarette smoking among patients in treatment for substance use disorders in France between 2010 and 2020. To our knowledge, this is the first study to provide systematic prevalence of cigarette smoking according to types of substances, namely alcohol, opioids, and stimulants, in France. The estimated prevalences are somewhat lower than those of prior studies conducted in the 1990s and 2000s, with prevalence ranging from 85% to 89%^18,19^. Several factors may explain such differences. First, the aforementioned studies focused on patients treated for alcohol disorders, whereas our study encompasses a wider range of substances, in line with differences observed in the UK^13^. Second, our study focused on daily smoking, discarding de facto current but non-daily smokers. Third, the lower prevalences may reflect, albeit with a time gap, the decreasing trend observed in the general adult population during the past decades, accompanied with the diffusion of electronic cigarettes^3^.

Nevertheless, the overall prevalence of daily cigarette smoking remains relatively high (72.7%, ranging from 69.9% in 2010 to 78.8% in 2020), at least twice as high as prevalences observed in the general adult population during the same time span^20^, in a similar proportion to other Western countries^9^. Tobacco use among individuals with SUD is endemic and, as such, regarded as a major public health concern^26^. Cigarette smoking among patients with SUD is no trivial issue, as smoking is associated with higher rates of dropping out of treatment^7^ and relapses^27^. Conversely, interventions for smoking cessation were found to increase the likelihood of long-term abstinence, for both alcohol and illicit substances^28^. Evidence suggests a mediation effect, in which tobacco cessation is a strong predictor of quality-of-life improvement, the latter enhancing substance abstinence^18^. The matter of tobacco and cigarette smoking among patients treated for substance disorders is even more critical given the low cessation rates^5,10,12^, significantly lower than those observed in populations without substance use disorders^29^.

Our results confirm certain similarities across substances: in the four cases considered in this study, females have lower odds of daily cigarette smoking, and smoke fewer cigarettes than males, as do younger patients, a tendency commonly observed in other countries^5^. People who use substances with psychiatric comorbidities are consistently more at risk to either smoke and smoke more, in line with prior results^30^. The number of cigarettes is negatively associated with ages of initiation, supporting the view of a generalized risk of substance (ab)use^31^ among older adults.

We uncover a mixed process: the increasing tendency of cigarette smoking observed over time, regardless of the types of substances inducing treatment, has been accompanied by a significant decrease in the average number of cigarettes smoked daily. The increasing trend of cigarette smoking observed here contrasts with recent data in the US^11^. We believe this increase to mirror a structural effect, namely the ageing population serviced in treatment centers, with tobacco use more widespread among older users. The results suggest that, although effective in the general population, prevention programs reach their limits when it comes to specific populations. It may also reflect increased marketing towards people more vulnerable to addictive behaviors^15,32^, to offset falling cigarette sales in the general population as tobacco use has somewhat increased in stigma during the past decade. Stigmatized patients are known to be more affected by difficulties in accessing care^33^, resulting in increased substance use. Moreover, unmet needs are likely to be amplified by the shortage of mental health professionals in disadvantaged communities^11^, nurturing a vicious circle that is yet to be broken.

The overall decreasing trend in the number of cigarettes per day occurs alongside the regular price increases in cigarette packs that took place during the past decades and the increasing use of electronic cigarettes^3^. However, strong disparities show among patients, with apparently paradoxically opposite trends regarding education level and occupational status. This discrepancy may be because each variable covers different, albeit complementary dimensions: the former encompasses health literacy (HL) whereas the latter refers to purchasing power. Similar to what has been observed in Western countries^34^, the overall decrease in tobacco use conceals significant disparities among subgroups, including patients treated for SUD. Particular attention has been paid to socioeconomic differences following an assumption of classic economics that posits health as a capital stock^35^, according to which health is an asset as well as a token of belonging to a social order in which tobacco smoking is poorly rated. Differential effects and the sensibility toward prevention messages are believed to be mediated by HL, e.g. the ability to perceive, process and understand health-related information, and the capacity to make adequate health decisions^36^; that is, a cognitive process mediating personal education, socioeconomic background and health-related outcomes^37^. Accumulated evidence suggests that the higher HL, the lower the propensity to smoke and, among current smokers, the higher odds to quit smoking^38^. Complementarily, lower HL is associated with increased likelihood to relapse in attempts to quit smoking^39^ and more severe related health negative outcomes. Our results suggest that HL holds within specific groups.

The idea that concomitant smoking among patients with SUD constitutes both a personal health hazard as well as a threat to their treatment outcomes, rather than an inconsequential collateral, is gradually gaining ground among healthcare teams^26^. Ironically, the ban on cigarette smoking in public places, and treatment centers in particular, is likely to disrupt their preventive efforts: since smoking is no longer permitted within the precincts, professionals may not be aware that a significant proportion of their patients do smoke. This, in addition to the previously mentioned sensitivity of patients to exposure to staff smoking^8^, adds an extra layer of concern in the global care pathway. Hence, tailored prevention toward patients is necessary but not sufficient *per se* to reduce smoking. Strengthening awareness and tobacco-related training among staff are also required^32^.

### Strengths and limitations

The study relies on a large sample and covers a full decade. Patients are surveyed by means of a standardized questionnaire. However, the repeated cross-sectional design of the survey prevents causation. Not all treatment centers provide date. A comparison of the available data sets with the annual active files (aggregated information transmitted to the Ministry of Health) suggested no significant differences regarding sociodemographics or the substances involved. Slight differences showed in terms of number of individuals (the non-participating centers were smaller and serviced fewer patients) as well as geographical location (non-participating centers are located in small urban and rural areas). The study focuses on cigarette smoking, discarding other types of tobacco products. The studied population encompasses patients serviced in treatment centers, whose profiles may differ from other patients treated by general physicians. The measures of cigarette smoking rely on self-report, with potential desirability bias, while the average number of cigarettes per day is subject to memory bias. The standardized questionnaire assumes smoking as an uninterrupted process and does not consider prior attempts to quit or relapses, nor does it take non-daily smoking into account. The study did not take race into account, as questions on ethnic origins are not permitted in France by law. Finally, occasional smoking was not taken into account, as well as the distinction between former and never smokers.

## CONCLUSIONS

In a context of general receding tobacco use in Western countries, cigarette smoking among patients in treatment for substance use disorders remains an unresolved issue calling for complementary interventions. On one hand, males, patients with education level and heavy smokers should be more specifically targeted. Given its generalization, electronic cigarettes and their concurrent use with tobacco cigarettes should be paid greater attention. On the other hand, enhanced awareness among staff is a growing necessity.

## Supplementary Material



## Data Availability

The data supporting this research cannot be made available for privacy or other reasons. The datasets generated and/or analyzed during the current study are not publicly available. The data contain sensitive information which allows the identification of individuals. It is therefore protected, and access can only be granted with special permission.
