# Supplementary material for: Daily cigarette smoking among inpatients for substance use disorders in France, 2010–2020: Commonalities and specificities across substances
Source: Tob Induc Dis. 2024 Nov 5;22:10.18332/tid/194097. doi: 10.18332/tid/194097 (PMC11536515; doi:10.18332/tid/194097)
Supplement: Supplementary file 1 [file TID-22-174-s1.pdf]

Supplementary table 1. Characteristics of patients treated for SUD, 2010-2020 (%)

		Alcohol	Opioids	Stimulants
Year of survey	2010	2.0	9.4	6.2
	2011	4.4	9.5	6.2
	2012	6.8	9.7	6.2
	2013	11.0	11.0	7.6
	2014	9.6	8.4	6.8
	2015	11.1	9.3	8.0
	2016	10.2	8.7	8.4
	2017	11.2	9.5	10.7
	2018	11.1	8.4	12.9
	2019	11.1	8.1	13.9
	2020	11.5	8.1	13.1
Gender	Males	79.0	77.6	78.5
	Females	21.0	22.4	21.5
Age	15-24 years old	5.1	8.6	12.1
	25-34 years old	19.1	35.7	35.3
	35-64 years old	75.8	55.7	52.6
Schooling	Incomplete secondary	63.0	72.5	58.1
	High school diploma	22.1	18.5	24.4
	Some college	14.9	9.0	17.5
Occupational category	Low	17.6	27.1	26.8
	Intermediate	68.8	65.7	59.1
	High	13.6	7.2	14.1
Smoking status	Daily	69.6	78.8	75.8
Age initiation: cigarette	16 years old or later	85.7	84.5	87.9
	<15 years old	14.3	15.5	12.1
Age initiation: substance leading to treatment	16 years old or later	81.7	92.5	92.1
	<15 years old	18.3	7.5	7.9
Severity: no dependence	Dependence	84.5	84.6	72.2
	No dependence	15.5	15.4	27.8
Length of treatment	0-5 months	23.2	12.6	25.5
	6-11 months	25.0	14.1	25.8
	12 months or more	51.8	73.3	48.7
Psychiatric disorders	None	73.8	65.3	68.4

	Anxiety/depression	13.3	14.8	14.5
	Other	12.9	19.9	17.1
N		**607122**	**283381**	**57189**

Source: RECAP survey.

© 2024 Janssen E. et al.
